# A Specific Melon Concentrate Exhibits Photoprotective Effects from Antioxidant Activity in Healthy Adults

**DOI:** 10.3390/nu10040437

**Published:** 2018-03-31

**Authors:** Laure Egoumenides, Audrey Gauthier, Sandy Barial, Marion Saby, Céline Orechenkoff, Guy Simoneau, Julie Carillon

**Affiliations:** 1 Bionov Research, 34090 Montpellier, France; laure.egoumenides@bionov.fr (L.E.); audrey.gauthier@bionov.fr (A.G.); 2 EA7288 Université de Montpellier, 34093 Montpellier CEDEX 5, France; sandy.barial@umontpellier.fr (S.B.); marion.saby@umontpellier.fr (M.S.); 3 Intertek France–Etudes Cliniques Paris, 75013 Paris, France; celine.orechenkoff@intertek.com; 4 Therapeutic Research Unit, Department of Internal Medicine, Lariboisière Hospital, 75013 Paris, France; guy.simoneau@lrb.aphp.fr

**Keywords:** ultraviolet radiation, oxidative stress, minimal erythema dose, sunburn cells, melanin, superoxide dismutase

## Abstract

Skin is the largest body organ and the first barrier to exogenous threats. This organ is constantly exposed to external factors such as ultraviolet radiation, which induces many adverse effects including sunburn, depigmentation, photo aging, photo immune suppression, and even skin cancer. Antioxidants seem to be good candidates in order to reduce ultraviolet-mediated damages and to prevent the health consequences of ultraviolet exposure. The present investigation aims to further characterize the potential skin photoprotective effects of a food supplementation and a topical administration of a melon concentrate alone or in combination. A clinical study assessing the Minimal Erythema Dose (MED) was first set up to evaluate photoprotection. Afterward, an independent in vitro study was performed on human skin explants from a donor to evaluate the effect of the melon concentrate at different levels including on the sunburn cells formation and on the endogenous antioxidant enzymes and its influence on melanin. Clinical study results demonstrate that melon concentrate application and/or supplementation increased MED. It also increased the endogenous antioxidant enzymes and reduced sunburn cells and melanin level on irradiated skin explants. Therefore, it is suggested that melon concentrate administration (oral and/or topical) could be a useful strategy for photoprotection due to its antioxidant properties.

## 1. Introduction

Human skin is the largest body organ. With its strategic location at the interphase between external and internal environment, the skin barrier has an essential role in detecting external factors and protecting, restoring, and maintaining body homeostasis [1,2,3].

Skin is exposed to external factors such as Ultraviolet radiation (UVR), which induces many adverse effects. UVR is part of the spectrum of electromagnetic radiation emitted by the sun and includes the wavelength range from 100 to 400 nm: UVC (100–280 nm), UVB (280–315 nm), and UVA (315–400 nm). Nearly 100% of the UVC and 90% of the UVB rays are blocked by the ozone layer in the stratosphere. Therefore, ambient sunlight is composed of UVA (90–95%) and UVB (5–10%) [1,4,5,6,7].

As the skin is directly exposed, it is the organ that is the most susceptible to be damaged by UV light. Exposure to UVR has both beneficial and adverse effects on human health. UVB are responsible for the production of vitamin D by direct conversion of 7-dehydrocholesterol into vitamin D3 [2]. This plays a critical role in the maintenance of calcium homeostasis in the body and in other important processes. In contrast, sun exposure causes acute and chronic changes to the skin. The main signs of photo damages are sunburn, photo immune suppression, photo dermatoses, depigmentation, photo aging, and cutaneous malignancy [1,8,9,10].

UVB is the major wavelength that causes sunburn [7]. Sunburn is a stereotypical inflammatory response induced by exposure to an erythemogenic dose of UVR. Sunburn is characterized clinically by redness, which is mediated by dermal vasodilatation, and edema, which is mediated by increased vascular permeability and inflammatory cell infiltration [10]. Histologically, it induces apoptotic keratinocytes called Sunburn Cells (SBC) among others harmful effects [6].

While UVB is mostly absorbed by several major cutaneous targets in the epidermis, UVA photons are only partly absorbed by the upper layers of skin and penetrates deeper into the dermis [11]. Actually, UVB is especially absorbed by DNA inducing covalent modifications such as the formation of the mutagenic 6–4 cyclobutane pyrimidine dimmers [1]. By contrast, UVA is responsible for the generation of Reactive Oxygen Species (ROS). Therefore, UVA can indirectly damage the DNA via the production of ROS [7,11]. Both direct and indirect DNA changes interfere with transcription and replication and render skin cells susceptible to mutagenesis as well as significantly increases skin cancer risk [1,6,12].

ROS can inappropriately activate signaling pathways, interfere with genome maintenance, and affect apoptosis [1,2,5,11]. Oxidative stress increases the production of metalloproteinases, which is a group of protease enzymes responsible for the degradation of components of the extracellular matrix such as collagen and elastin fibers. This is therefore linked to age-related loss of skin elasticity and subsequently to photo aging [7,13]. Photo-oxidative stress can also disturb cell signaling, proinflammatory processes, and nociceptive processes and promotes photocarcinogenesis and photo ageing through Lipid peroxidation (LPO) [2].

Furthermore, UV exposure initiates a cascade of inflammatory cytokines via activation of the AP-1 and NF-kB pathways [7,11]. Moreover, several studies have demonstrated the immunosuppressive effects of UVB and UVA and their implication in skin cancer formation [5,8,14,15].

Since skin acts as the first barrier against UVR, it has developed an innate adaptive tanning response using melanin as a chromophore so as to protect the skin against further UV insult. Cellular damage response activation induces melanin production by melanocytes and melanin deposition in keratinocytes, which generates an enhanced pigmentation of the skin [1,8]. However, although skin pigmentation assists in the attenuation of the photo-damaging effects, melanin is not entirely protective against UV rays [5,8]. Knowing the harmful effects of UV exposure, it is important to provide the skin with an additional protection. Beyond UV filters and physical photoprotective agents, antioxidants seem to be good candidates in order to mitigate UV-mediated damage and to prevent the health consequences of UV exposure. Interestingly, it was previously reported that oral supplementation with a specific melon concentrate increased endogenous antioxidant defenses and reduced oxidative stress in several targeted organs [16,17,18,19]. However, this melon concentrate has never been tested using another route of administration. Therefore, we don’t know yet if a topical application will induce the same antioxidant effects as oral supplementation.

As such, the present investigation was designed to further characterize the potential skin photoprotective effects of a food supplementation and a topical administration of a melon concentrate alone or in combination. After a clinical study assessing the Minimal Erythema Dose (MED) as a way to evaluate photoprotection, another independent in vitro study was set up on human skin explants from a donor in order to evaluate the effect of the melon concentrate on the SBC formation, the endogenous antioxidant enzymes level, and the influence on melanin.

## 2. Materials and Methods

### 2.1. Preparation and Characterization of the Melon Concentrate

SODB^®^ (Bionov, Avignon, France) is a dried melon juice concentrate particularly rich in superoxide dismutase (SOD), which results from a patented process. Briefly, the pulp of a specific proprietary and non-GMO melon variety (*Cucumis melo* L.) is separated from skin and seeds and crushed before centrifugation. Then the melon juice undergoes filtration and concentration steps. Lastly, the obtained melon juice concentrate is freeze-dried. For oral administration, this dried melon juice concentrate is coated with palm oil in order to preserve its activity from the digestive enzymes secreted above the small intestine. For topical application, it is coated with modified starch microspheres in order to protect its activity from exogenous factors and particularly from an aqueous environment. Detailed information about the antioxidant content of this melon concentrate has been previously published [20]. In this study, it contains 14 U SOD/mg powder measured according to the method of Zhou and Prognon [21]. In the clinical study part, volunteers were given one small hard capsule per day. One capsule contains 20 mg of SODB (280 U of SOD), excipients for the active supplement, and excipients only for the placebo. The active cream used for clinical and in vitro experiment was formulated with neutral vegetal oil, ultrapure water, surfactant, and thickener and applied at 12 U of SOD per cm^2^ of skin in both clinical and explants studies. Placebo cream did not contain SOD in its formulation.

### 2.2. Clinical Study Design

The clinical trial was an intervention study of sun protection properties of the melon concentrate based on the individual evaluation of MED at different times. The study was set up at Intertek France. It was a monocentric study performed from April, 2017 to July, 2017. The protocol followed was a controlled clinical study vs. placebo, randomized, double blind.

The inclusion criteria were to be between 18 to 50 years old and in full health, to be Caucasian with skin type II and type III, and have an Individual Typology Angle (ITA) included between 28° and 55° on the buttocks and have a uniform skin color on the buttocks. Other criteria were not to take any drugs, dietary supplements, or topical products and not to change their daily routine especially concerning sun exposure and diet. Subjects who have given written informed consent and are willing to comply with the study requirements are included in the study.

The exclusion criteria were to be pregnant or breast-feeding, to have a past history of phototoxic, photoallergic or other abnormal responses to sunlight, to have allergies or sensitivity to cosmetic products, toiletries, sunscreens, topical drugs and/or melon or any compounds of the administered products, to have a pre-existing or dormant dermatologic conditions, to be on a chronic medication, to have systemic treatment which may modify the cutaneous state on the day of inclusion or finished in less than 15 days from the inclusion visit or during the month preceding the inclusion visit, to have a sunburn, suntan, scars, active dermal lesions and/or even tones on the buttocks area, to have had sun exposure on the buttocks area for at least four weeks prior to study inclusion, to have used a tanning product on the buttocks area in the two weeks prior to study inclusion, and to be accustomed to using tanning beds. Patients who took a dietary supplement during the last month are excluded. These volunteers provided a written informed consent.

**Figure 1:**
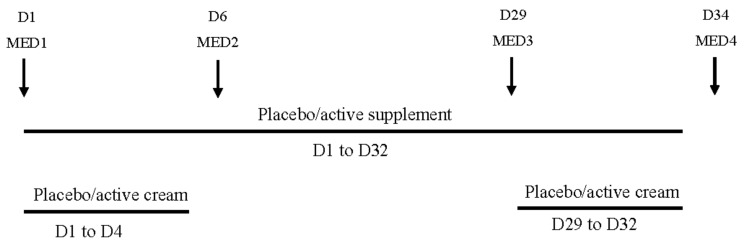
Schematic representation of the clinical study design. D**:** day; MED: Minimal Erythema Dose.

The volunteers were assigned by randomization into four groups of 22 subjects each. One group received the active cream and the placebo complement (active cream group), one group received the active cream and the active supplement (double active group), one group received the placebo cream and the placebo complement (double placebo group) and the last group received the placebo cream and the active supplement (active supplement group). The active cream and placebo cream were applied during two cycles of four days starting from day 1 (D1) to D4 and from D29 to D32. Creams were applied by qualified technicians at the rate of 2 mg/cm^2^ on four test sites of 35 cm^2^ (two sites on each buttock). The placebo and active supplement were taken daily (one capsule at breakfast) at home by volunteers during 32 days from D1 to D32. The capsules and creams were administered or applied in a double-blind approach. The clinical study design is schematized on Figure 1.

#### Minimal Erythema Dose Measurements

In this clinical study, MED was evaluated four times during a visit to the study center. The assessments of the intensity of the erythema were visually determined by a unique trained and experienced assessor on the different sites. It was a blind determination. MED1 corresponds to the baseline measure before the start of topical cream application and food supplementation. MED2 at D6 is an intermediate measure after four days of cream application. MED3 at D29 is an intermediate measure before the second cycle of topical application. Finally, MED4 at D34 is the final measure, corresponding to the end of the second topical cream application and food supplementation.

MED is evaluated in order to determine the photoprotective effect of the cream application or food supplementation. It is defined as the amount of UV radiation that will produce MED (sunburn or redness caused by engorgement of capillaries) of an individual’s skin within a few hours following exposure [22]. The source of radiations was an ORIEL solar simulator (Newport, Stratford, CT, USA) and the UV light was comprised between 280–400 nanometers (UVA and UVB). The dose of UV radiation used to determine the baseline MED depends on the ITA of the subject, on the exposed zone (buttocks, back or arms), on the exposition period of the year, and on the subject’s phototype as well as an interpersonal parameter. Two subjects with the same ITA can have different MED values. Therefore, different UV doses were tested so as to get the more accurate MED determination. In order to determine the MED value, six skin areas of 1 cm^2^ were exposed to increasing doses of UV radiation (six doses were chosen with a 15% increasing scale). 16 to 24 h after the end of the UV exposition, the erythema visual scoring was realized on each of the skin’s area. The erythema peak was obtained 16 h after the UV exposure. Then the erythema started to decrease 24 h after the exposure.

The scale used to characterize the reaction and determine the MED start from 0. There was no reaction to 3 where strong erythema with edema was obtained. The MED corresponds to the first UV dose for which we obtained a visual score of 1, showing mild but definite erythema.

Therefore, the higher the MED value is, the higher UV dose is needed to make an erythema appear with a visual score of 1. Therefore, a product with a photoprotective effect will increase the MED.

### 2.3. Skin Explants Experimental Design

Skin explants were obtained with the informed consent of a patient (woman, 31 years old, Caucasian type) who underwent abdomen reduction surgery. Fat tissues were removed from the skin and the explants were cut (2.0 cm^2^). They were kept under cultivation in contact with the culture medium at 37 °C in 5% CO_2_ humidified air. The culture medium consists of Dulbecco’s Modified Eagle Medium (DMEM) supplemented with 2 mM of l-glutamine, 50 U/mL of Penicillin, and 50 µg/mL of streptomycine, 10% Fetal Bovine Serum (FBS), and 0.4 µg/mL of Hydrocortisone. Active and placebo cream were applied on the surface of the explants. Explants were pre-incubated over 72 h with a topical application renewal every day. Untreated control was also run in parallel. Following this pre-incubation step, explants were treated again (except the non-treated control) and incubated for 2 h. Explants were then irradiated with UVB at 1.5 J/cm^2^ and UVA at 22 J/cm^2^. This step was performed using a natural light simulator SOL500 equipped with a H_2_ filter (Dr. Hönle, AG, Gräfelfing, Germany) and explants were then incubated again. Non-irradiated control was protected from the light during the irradiation. Three independent experiments were performed on five explants. Afterward, different samples were collected 24 h after irradiation. On one hand, explants were harvested, fixed in formalin, embedded in paraffin, and the histology methods were set up. On the other hand, explants were snapped frozen for further Western Blot analysis.

#### 2.3.1. Histology

Paraffined sections (5.0 μm) were stained with Hematoxylin Eosin Safranin (HES) for SBC detection. Changes in epidermal morphology were followed by qualitative analysis using NIKON E400 optical microscope with the camera NIKON DS-Ril (Nikon instruments, Champigny sur Marne, France) connected to the computer with the software NIS element 4.13.04 (Nikon instruments Champigny sur Marne, France) for capturing images. SBC were identified thanks to different characteristics after staining. Dark pyknotic and condensate basophilic nucleus, eosinophilic cytoplasm, and intracellular space formation. SBC were counted and reported to the epidermal surface. Cell counting was performed on five selected sections per blade allowing 15 different values per conditions.

For melanin quantification, paraffined section of 5 µm were stained using the Fontana Masson method. After staining, melanin was colored in black while nuclei were colored in red. Therefore, melanin was measured in quantity in five to ten given fields. All analyses were performed using image analysis software (ImageJ).

#### 2.3.2. Western Blot Analysis

The protein extraction was carried out on ice in 20 mM of Tris buffer (pH 6.8) containing 150 mM sodium chloride, 1 mM EDTA, 1% Triton 20%, 0.1% SDS, and 1% protease inhibitor cocktail (Sigma-Aldrich). After centrifugation (1500 rpm, 15 min at 4 °C), the supernatant was collected and the extracted tissue proteins were then separated by SDS polyacrylamide gel electrophoresis. Equal amounts of proteins were loaded onto a 15% acrylamide gel with a 4% stacking acrylamide gel. Migration was conducted in a Tris-glycine-SDS buffer. After separation, proteins were transferred onto nitrocellulose membranes (Whatman, Dassel, Germany).

Proteins were detected by Western blot analysis. The primary antibodies against human Superoxide Dismutase (SOD) Cu/Zn-SOD, Mn-SOD, glutathione peroxidase (GPx), catalase (CAT), and the control protein glyceraldehyde 3-phosphate dehydrogenase (GAPDH) were purchased at R&D Systems (Lille, France) or Sigma-Aldrich. Expression of GAPDH was used for checking the equal protein load across gel tracks. Secondary antibodies (Sigma-Aldrich, Saint-Quentin Fallavier, France), coupled with horseradish peroxidase were used for revealing the primary antibodies. Band densities were obtained by scanning the membranes. Image analysis (ImageJ) was used to quantify after standardization within membranes by expressing the density of each band of interest relative to that of GAPDH in the same lane. Results are then expressed as a percent of values obtained in unirradiated or irradiated skin explants.

### 2.4. Statistical Analyses

The clinical data points were expressed as means ± SEM. The changes between D1 and D6, D30, and D34 were tested for each group using repeated measures ANOVA with subjects as random factor and the time as repeated measures. Pairwise comparison between times was performed using the Dunnett test. The intergroup comparison was carried out on each difference D6-D1, D30-D1, and D34-D1 using the one-way ANOVA with the treatment as a fixed factor. The pairwise differences between the 4 treatments were tested using a two-step analysis. First, each active treatment was compared with the reference treatment with the double placebo using the Dunnett test and then the active treatments were compared between them by using the Tukey test.

Regarding non-clinical results, values were presented as means ± SEM. Data were tested using one-way ANOVA followed by protected least significant difference tests.

Statistical analyses of the data were carried out using SPSS 24.0 software (IBM, Armonk, NY, USA). The *p*-values that were less than 0.05 were considered to be significant.

## 3. Results

### 3.1. Clinical Study Results

#### Study Population

Ninety three volunteers aged between 19 and 50 years (mean 37.2) were recruited and randomly assigned into four test groups. Five subjects left the study during its progress and were excluded from the study. Therefore, the study was conducted with four test groups including the placebo group (*n* = 22), the active cream group (*n* = 22), the active supplement group (*n* = 22), and the double active group (*n* = 22). The gender-ratio was 84.1% women. There were no statistical differences between the four groups at baseline.

### 3.2. Melon Concentrate Administration Increased Minimal Erythema Dose

Table 1 shows MED measurements before, during, and after melon concentrate application and/or supplementation.

Intragroup analysis showed that there was no significant change over time for the double placebo group (*p* = 0.5167). When compared to D1, no significant differences were found at D6(*p* > 0.05), D30 (*p* > 0.05), and D34 (*p* > 0.05). By contrast, a significant effect over time was highlighted for each active treatment (*p* < 0.0001). Regarding active cream topical application, compared to D1, MED was significantly increased by 17.5% at D6, after four days of active cream topical application (*p* < 0.0001). The difference was not significant at D30 i.e., at the start of the 2nd cycle of application (*p* > 0.05), but the MED was significantly increased by 20.1% at D34 after four days of application(*p* < 0.0001). Concerning the active supplement group, compared to D1, the change in MED was not significant at D6 after six days of treatment (*p* > 0.05) but the MED was significantly higher at D30 (+17.3%, *p* < 0.0001) and D34 (+17.8%, *p* < 0.0001) after 30 and 32 days of treatment, respectively. Lastly, for the active cream and active supplement combination group, when compared to D1, MED was higher after six days of treatment (D6) but the difference (+11%) was not significant (*p* = 0.07) and MED was significantly increased by 20.8% at D30 (*p* = 0.0003) and by 28.2% at D34 (*p* < 0.0001).

**Figure 2:**
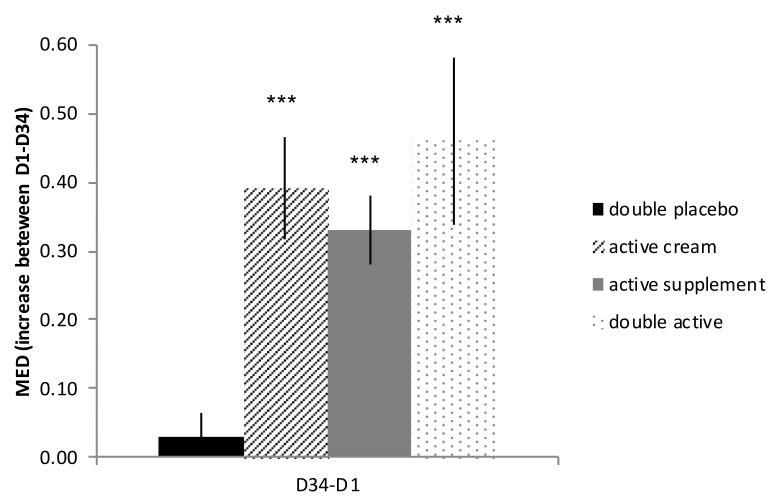
Influence of melon concentrate on Minimal Erythema Dose (MED) during clinical study. Results are presented as MED differences between day 1 (D1) and day 34 (D34) mean ± SEM. *** *p* < 0.001 effect of melon concentrate treatment compared with the placebo group.

With reference to intergroup analysis, regarding the increase between D1 and D6 (D6-D1), there was no significant difference between the supplement group and the placebo (*p* > 0.05) while the MED was significantly higher for the treatment with cream (*p* < 0.0001) and with the double active treatment (*p* = 0.0057). At the end of treatment (D34), as presented on Figure 2, the MED was increased in each actively treated group when compared to placebo (D34-D1). The increase in MED was significant for the supplement (*p* = 0.0205), cream (*p* = 0.0036), and double active treatment (*p* = 0.0005).

Finally, no significant difference was highlighted between the three active treatments. At the end of treatment, the MED increase was higher with the combined treatment than the single treatment with the cream or the supplement but the difference was not significant.

### 3.3. Human Explants Results

#### 3.3.1. Topical Application of Melon Concentrate Reduced the Number of SBC on Irradiated Human Skin Explants

**Figure 3:**
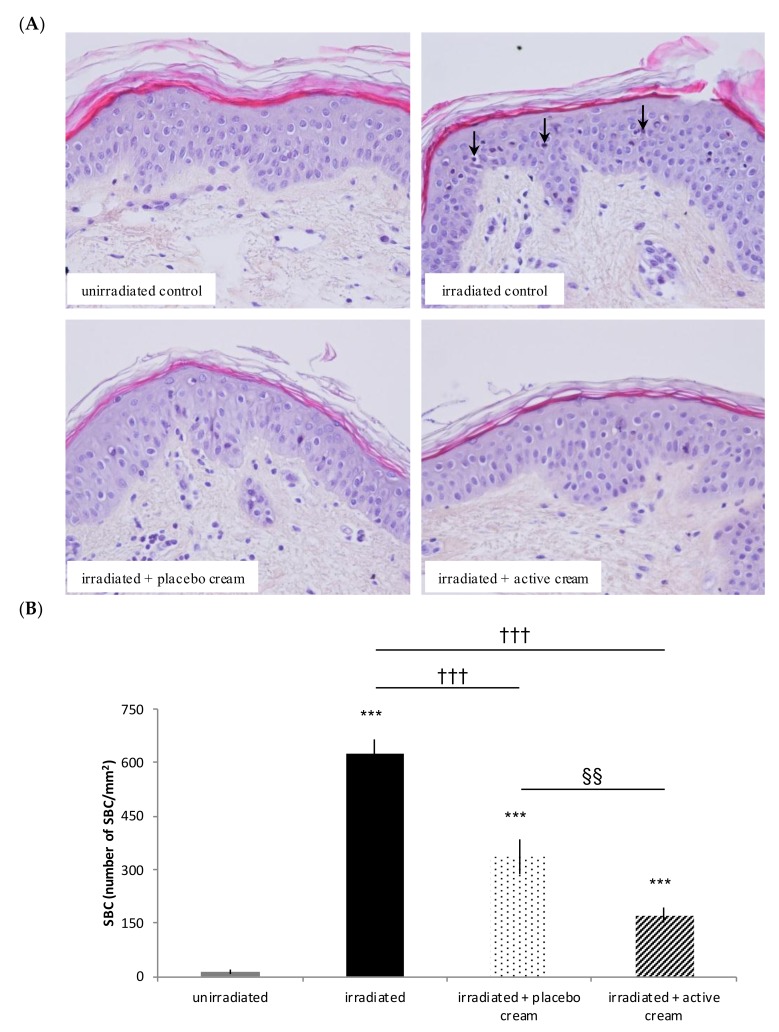
Influence of melon concentrate application on the formation of Sun Burn Cells (SBC) after irradiation of human skin explants. (**A**) HES histochemical staining of human skin explants for SBC observation. Arrows show SBC. (**B**) SBC were quantified after histochemistry analysis on five transverse sections per skin explant. Results are expressed in SBC/mm^2^ of skin. *** *p* < 0.001 compared with unirradiated skin explants, ††† *p* < 0.001 compared with irradiated skin explants, and §§ < 0.01 effect of melon concentrate treatment compared with the placebo group.

As shown in Figure 3, the number of SBC on human skin explants was significantly increased after irradiation compared to unirradiated explants. Additionally, the number of SBC on irradiated skin explants was significantly decreased by 72.5% after topical application of the melon concentrate when compared to irradiated untreated explants. A placebo cream effect was observed when compared with irradiated untreated explants (*p* value < 0.05). However, a significant decrease of SBC is obtained using the active cream when compared with the placebo cream.

#### 3.3.2. Topical Application of Melon Concentrate Reduced the UV-Induced Oxidative Stress on Human Skin Explants

**Figure 4:**
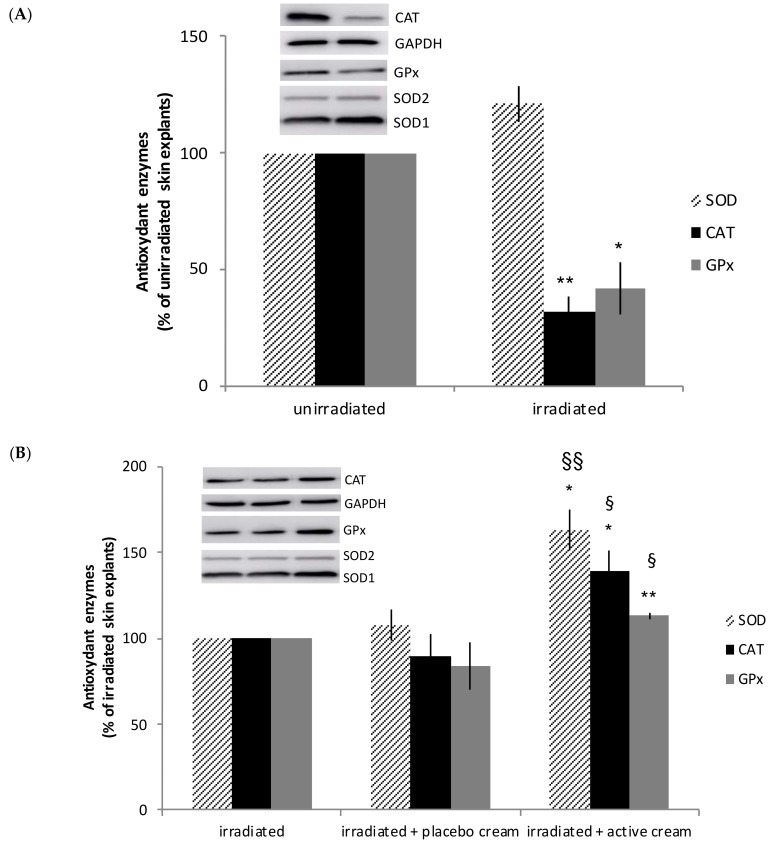
Influence of melon concentrate application on the antioxidant enzymes level after irradiation of human skin explants. Quantification was made after standardizing within membranes by expressing the density of the band of superoxide dismutase (SOD), catalase (CAT), and glutathione peroxidase (GPx) relative to that of glyceraldehyde 3-phosphate dehydrogenase (GAPDH) in the same lane. Results are then expressed as (**A**) relative change from un-irradiated skin explants band intensity and (**B**) relative change from irradiated skin explants band intensity. (**A**) ** *p* < 0.01 and * *p* < 0.05 compared with unirradiated skin explants. (**B**) §§ *p* < 0.01 and § *p* < 0.05 compared with irradiated skin explants, ** *p* < 0.01 and * *p* < 0.05 effect of melon concentrate treatment compared with the irradiated + placebo cream group.

As presented in Figure 4A, the level of CAT and GPx measured by western blot analysis was decreased after irradiation of human skin explants. While no effect was observed after applying the placebo cream, skin SOD, GPx, and CAT were significantly increased by 63%, 39%, and 13%, respectively, in irradiated skin explants after topical application of the active cream when compared to untreated explants (see Figure 4B).

#### 3.3.3. Topical Application of Melon Concentrate Modulated the Melanin Level on Irradiated Human Skin Explants

**Figure 5:**
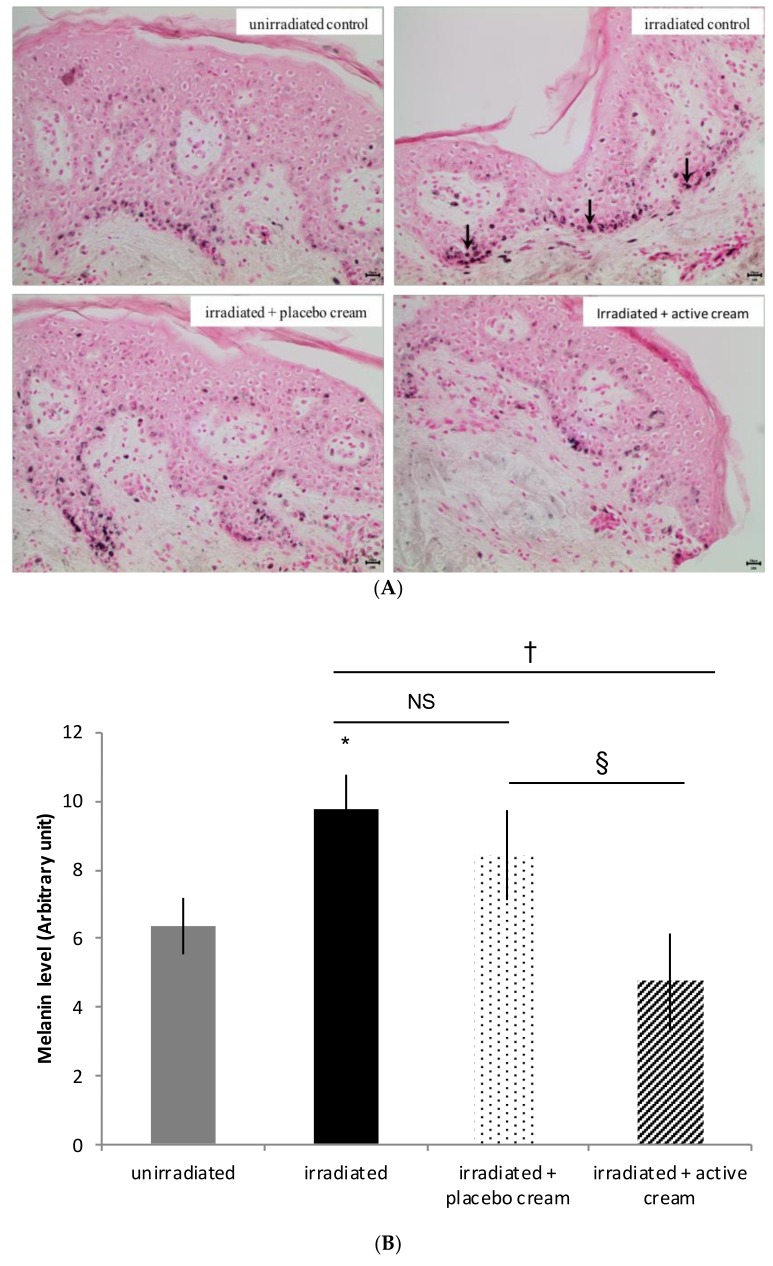
Influence of melon concentrate application on the melanin level after irradiation of human skin explants. (**A**) Fontana Masson histochemical staining of human skin explants for melanin observation. Arrows show melanin; (**B**) Melanin level determined after histochemistry analysis on at least 30 transverse sections per skin explant. Relative levels were presented in arbitrary units as mean ± SEM. * *p* < 0.05 compared with unirradiated skin explants, † *p* < 0.05 compared with irradiated skin explants, § < 0.05 effect of melon concentrate treatment compared with the irradiated + placebo cream group. NS: non-significant.

As presented in Figure 5, the level of melanin was significantly higher (by 53%) after skin explants irradiation in comparison with unirradiated skin explants. After irradiation of human skin explants and active cream application, there was no difference in the melanin level when compared with the un-irradiated skin explant group. No significant difference was observed with the placebo cream application.

## 4. Discussion

The clinical trial aimed to assess the photoprotective effect of a melon concentrate topical application or oral supplementation on healthy people with skin type II and skin type III. There is no placebo effect observed since there is no significant modification of the MED in the double placebo group over time. Our results clearly show a significant increase of MED after melon concentrate application and supplementation compared to the placebo group. The increase of MED pointed out a photoprotective effect of the melon concentrate on the skin. Our results align with other studies showing a photoprotective potential of plant products such as polyphenol from green tea or grape seeds and other antioxidants including vitamin C, E, or ubiquinone [23,24,25]. While the MED increase is obtained after four days of topical active cream application and the equivalent MED increase is obtained after a longer period of food supplementation. However, no measurements were performed between D6 and D30, which means it would be necessary to add intermediate MED measurements in order to have a more precise idea of the timing of efficacy of the active supplement. No significant difference was obtained at D30 when we compare the active cream group with a placebo group between the two cycles of topical treatments. Therefore, there is no long-time effect of the active cream. The fastest efficacy of topical cream application is in agreement with its direct action on the skin as a targeted organ. This allows for a faster action because it is more specific and localized. Oral, in comparison with topical administration, provides the added benefits of systemic distribution [8]. When the active cream and active supplement were used in combination, there was a tendency showing that the MED was increased even better than with the products used alone. However, no significant difference was highlighted between the products used alone or in combination. Therefore, it would be of interest to further study combination of oral supplementation with a topical application treatment of longer duration and a larger sample size in order to expect an increased skin photoprotective effect. Because of those promising results, further experiments were carried out using skin explants in order to better understand the mechanism involved in the observed skin photo-protection.

Regarding the other independent in vitro experiment on human skin explants, we observed that UV radiation exposure increased the number of SBC on a human skin explants model. This particular result totally matches with the literature and the normal response described in many prior studies [10,26]. In addition, UV radiation induces an inflammatory response of the skin. Sunburn inflammation causes vasodilation of cutaneous blood vessels, which results in the erythema and chemical mediators being released. This leads to infiltration of neutrophils and T lymphocytes and both damaged keratinocytes (SBC) while Langerhans cells undergo apoptotic changes [14,27,28]. Our results also show evidence that the number of SBC was reduced on irradiated human skin explants after topical application of melon concentrate. The anti-inflammatory activity of some antioxidants has been well documented and, therefore, we can hypothesize that the melon concentrate has anti-inflammatory effects and reduces the UV-induced cytotoxicity [29,30,31].

It is demonstrated in several studies that UV radiations increase the number of ROS [32,33,34]. At a low level, ROS are essential for the normal course of aerobic metabolism. However, a high level of those short-lived entities create oxidative stress and can induce many adverse effects from DNA damage to skin cancer [1,8,9]. Presently, we observe in the in vitro study that irradiation of human skin explants decreases the level of CAT and GPx, which are natural antioxidant enzymes preventing the accumulation of ROS. This aligns with other study results. Indeed, CAT, SOD, and GPx levels and activities are reduced in the HaCaT in vitro model and in Human Dermal Fibroblast cells after irradiation [32,35]. As a result, the increase of ROS after irradiation can be correlated with the fact that the level of antioxidant enzyme decreases after UV irradiation of human skin explants. After topical application of melon concentrate on human skin explants, the level of SOD, CAT, and GPx is significantly increased in comparison with placebo and irradiated skin. This allows restoring the UV-induced decrease of antioxidant enzymes level. Those results are consistent with the data obtained in previous studies. We demonstrated that oral melon concentrate administration was associated with an increase in the endogenous antioxidant defense in animals [16,17,18,19,20,36,37,38]. We have shown that melon SOD concentrate supplementation could increase endogenous antioxidant enzymes (SOD, GPx, CAT) in the liver and adipose tissue of obese hamsters [17,18], in the heart of spontaneously hypertensive rat [19], and oviducts of hens [36]*.* We demonstrated in the clinical part that both topical application and oral supplementation of the melon concentrate induced a photoprotective effect on the skin by increasing MED. Therefore, even if experimental conditions are not exactly the same, we hypothesize that beneficial effects observed in this clinical study may be due to the enhancement of endogenous antioxidant enzymes that were found using skin explants. However, further investigations need to be done in order to confirm this hypothesis and the precise mechanism of action remains to be determined.

We observe an increase of melanin quantity in the basal epidermis after UV irradiation of human skin explants during the in vitro experiment. Literature describes two types of responses to UV. First, UV radiation induces oxidation of the pre-existing epidermal melanin and a redistribution of melanosomes to the epidermal upper layer, which induces an immediate skin darkening. Second, delayed skin pigmentation appears. It is characterized by an up-regulation of melanin synthesis by inducing an increased level of epidermal melanin and, therefore, providing photoprotection [4,39]. In this case, human skin explant samples were paraffin embedded 24 hours after UV irradiation. We can, therefore, suppose we were at the beginning of the UV-induced melanogenesis as we observe an increase of melanin in the basal epidermis. Afterward, we hypothesize that melanin synthesis would have continued. Melanin would probably be transported to keratinocytes nuclei via melanosomes in order to protect cells from UV-induced damages. After melon concentrate application, the level of melanin in the irradiated skin explants is close to the level of melanin in non-irradiated skin explants. Therefore, our theory is that melon concentrate corrected the melanin level change induced by UVR. Adaptive melanization is a complex physiologic response involving multiple skin cell types interacting in a variety of ways. In addition, besides functioning as a broadband UV absorbent, melanin has also antioxidant and radical scavenging properties [4,29,34,40,41]. Therefore, we can hypothesize that application of the melon concentrate on skin prevents ROS imbalance by inducing an increase of endogenous antioxidant enzymes. This reduces the inflammatory response induced by direct UV radiation [30,31] and indirectly reduces the formation of SBC. As skin is partly protected from UV radiation following melon concentrate application, the natural protecting tanning response should be reduced. That is why there is no difference between the melanin levels in non-irradiated skin and irradiated skin after the melon concentrate is applied. As described in other studies using antioxidant compounds, we can suppose that the melon concentrate allows a biological protection by attenuating UV-induced cytotoxicity and inflammatory responses, which reduces UV-related over-production of ROS and loss of endogenous antioxidants [13,32,34,42]. The added value of the melon concentrate is that it does not only act as a scavenger of ROS but it also has the ability to stimulate the endogenous antioxidant system in order to induce a more robust antioxidant effect. In addition, it is important to confirm the results with other donors to be more representative of the population. Skin explants as well as animal models were used to answer other problematics and have confirmed the endogenous antioxidant results and the reproducibility of the skin explant technique (data not shown) [16,17,18,36,38]. The precise mechanism of action of the melon concentrate remains to be clarified and warrants further investigation. Even though the melon concentrate contains a high level of SOD, a direct effect of the enzyme is not considered because its high molecular weight excludes intestinal absorption. This current study aimed to analyze the effect of the melon concentrate on the skin for the first time and skin absorption following topical application is excluded for the same reason. Currently, this particular melon concentrate is thought to trigger a cascade of events that initiates the induction of antioxidant defense in various tissues from the intestine or the skin through a local stimulation of the immune system [16,17,18,36,38,43]. The natural product contains active ingredients that will activate an antioxidant defense of the host with a systemic effect at multiple organ levels following oral administration.

In conclusion, melon concentrate application and/or supplementation have beneficial effect on the skin i.e., increase of MED during the clinical study, increase of endogenous antioxidant enzymes, reduction of SBC, and melanin level on irradiated skin explants. This photoprotective effect is obtained thanks to antioxidant properties of the melon concentrate. UV is one of the well-known causes of oxidative stress, which is involved in aging and many disorders. Knowing the beneficial effect of the melon concentrate in photoprotection, it could be of interest to further investigate its possible beneficial effects on the skin such as reducing skin aging, healing, or depigmentation, which are issues related to ROS imbalance.

## Figures and Tables

**Table 1:** Minimal Erythema Dose measurements before, during, and after melon concentrate application and/or supplementation.

	Double Placebo Group	Active Cream Group	Active Supplement Group	Double Active Group
MED at D_1_	1.91 ± 0.13	1.94 ± 0.14	1.85 ± 0.14	1.63 ± 0.10
MED at D_6_	1.91 ± 0.13	2.28 ± 0.19 ***	1.88 ± 0.14	1.81 ± 0.11
MED at D_30_	1.93 ± 0.12	1.98 ± 0.17	2.17 ± 0.17 ***	1.97 ± 0.15 **
MED at D_34_	1.94 ± 0.12	2.33 ± 0.19 ***	2.18 ± 0.16 ***	2.09 ± 0.17 ***

Values are presented as mean ± SEM. ** *p* < 0.01, *** *p* < 0.001 effect of the melon concentrate compared with placebo. MED: Minimal Erythema Dose.
